# Bile acid–microbiota interactions in cardiometabolic diseases: mechanisms and emerging therapeutic approaches

**DOI:** 10.3389/fmicb.2025.1689026

**Published:** 2025-12-17

**Authors:** Feiyu Chen, Lihong Gong

**Affiliations:** 1 Liaoning University of Traditional Chinese Medicine, Shenyang, Liaoning, China; 2 Affiliated Hospital of Liaoning University of Traditional Chinese Medicine, Shenyang, Liaoning, China

**Keywords:** bile acid metabolism, FXR, TGR5, atherosclerosis, metabolic syndrome, cardiovascular and cerebrovascular disease

## Abstract

The gut microbiota and bile acids co-regulate host metabolism through bidirectional interactions. This interaction critically influences the pathogenesis and progression of cardio-metabolic diseases (CMDs), which include diabetes, obesity, non-alcoholic fatty liver disease (NAFLD), and cardiovascular diseases. Growing evidence establishes bile acid metabolism as fundamental to the pathogenesis of CMDs. Bile acids activate both the nuclear receptor FXR and the membrane receptor TGR5, which in turn influence glucose and lipid metabolism, modulate inflammatory processes, and affect vascular functions. These signaling pathways collectively link metabolic and immune networks within the cardio-metabolic axis. This review provides an integrative overview of recent findings in bile acid signaling and its cross-talk with metabolic and immune pathways in CMDs. It critically evaluates disease mechanisms, discusses therapeutic candidates targeting bile acid pathways, and highlights future directions for the precise management of metabolic-immune disorders.

## 1 Introduction

Cardiometabolic diseases (CMDs), comprising obesity, type 2 diabetes mellitus (T2DM), nonalcoholic fatty liver disease (NAFLD), and atherosclerotic cardiovascular disease (ASCVD), represent a rapidly increasing public health challenge worldwide. More than 537 million individuals are affected by T2DM globally; NAFLD is present in 25 to 30% of the population, and ASCVD is responsible for nearly a third of global deaths (Gillard and Leclercq, 2024). Emerging evidence implicates gut microbiota dysbiosis and its metabolites, especially bile acids, as central mechanistic links in CMD pathogenesis, modulating host metabolism and inflammation via FXR and TGR5 signaling pathways (Gillard and Leclercq, 2024; Collins et al., 2023). Bile acids, the end products of cholesterol metabolism, play pivotal roles in lipid emulsification and maintaining systemic metabolic balance (Li and Chiang, 2024). By activating FXR and TGR5 receptors, bile acids regulate lipid metabolism, glucose homeostasis, and energy expenditure, thereby influencing the pathophysiology of atherosclerosis, hypertension, and heart failure (Biagioli and Fiorucci, 2021). Hepatocytes synthesize and secrete bile acids, maintaining whole-body cholesterol balance. Conversion of cholesterol to bile acids reduces NAFLD and cardiovascular risks (Fuchs, 2022). Critically, bile acids and gut microbiota exhibit bidirectional crosstalk: microbial shifts alter bile acid synthesis, enterohepatic circulation, and receptor signaling (Collins et al., 2023).

Bile acid sequestrants reduce serum LDL-C, attenuating atherosclerosis. Pharmacological activation of receptors—notably FXR and TGR5—further suppresses hepatic cholesterol neogenesis and ameliorates dyslipidemia (Jadhav et al., 2018). These observations highlight bile acid pathways as promising targets for cardiovascular interventions (Mahajan et al., 2023). Comprehending the multifaceted contributions of bile acids to CMDs and devising therapeutic strategies targeting these pathways are imperative for developing innovative metabolic treatments. Ongoing investigations continue to refine our understanding of bile acid biology, revealing novel molecular targets for clinical exploitation (Sonne, 2021).

Recent comprehensive reviews emphasize that gut microbiota regulates host immunity via bile acid metabolism, creating a critical interface between intestinal ecology and systemic metabolic balance. Dysbiosis in this axis is a key factor in CMD pathogenesis, including obesity and NAFLD (Varanasi et al., 2025).

In summary, the bidirectional interaction between gut microbiota and bile acids constitutes a core pathological link in CMDs. To gain a deeper understanding of this axis, the following section will systematically elaborate on the regulatory network governing bile acid metabolic homeostasis, from synthesis to enterohepatic circulation.

## 2 Regulation of bile acid metabolic homeostasis: from synthesis to the enterohepatic circulation

### 2.1 Enterohepatic circulation and FXR-mediated feedback regulation

In the liver, cholesterol is converted by CYP7A1 and related enzymes into primary bile acids (cholic and chenodeoxycholic acids), then conjugated with glycine or taurine and secreted into bile (Dong et al., 2025). After their synthesis, bile acids enter a highly efficient recycling process known as the enterohepatic circulation, which is critically modulated by the gut microbiota. About 95% of bile acids secreted into the intestine are reabsorbed in conjugated form in the distal ileum through the apical sodium-dependent bile acid transporter (Hofmann, 2009). These reabsorbed bile acids return to hepatocytes via the portal vein for resecretion into bile canaliculi. Termed enterohepatic circulation, this process recurs approximately six times daily in humans, enabling efficient bile acid recycling (Deng and Bae, 2020). The minor fraction escaping reabsorption undergoes fecal excretion, with minimal renal elimination, collectively preserving a total bile acid pool of ∼3 g within the hepatointestinal system. Gut microbiota perturbations can substantially modify bile acid composition, subsequently modulating host metabolic and immune responses (He Y. et al., 2025). This regulation mainly relies on gut microbiota-encoded bile salt hydrolase (BSH)—which catalyzes the deconjugation of conjugated bile acids to free forms, affecting bile acid pool composition and FXR/TGR5 signaling; CMD patients often have reduced BSH activity due to decreased BSH-producing microbiota, exacerbating metabolic disorders, and targeting BSH (Dong et al., 2025).

Bile acid metabolism is tightly controlled by feedback mechanisms. Farnesoid X receptor (FXR), a key intracellular bile acid sensor, monitors bile acid levels and dynamically regulates their synthesis and transport (Chiang and Ferrell, 2022). FXR further coordinates metabolic pathways in the liver and intestine to maintain systemic metabolic balance. The functional interplay between FXR and the G protein-coupled bile acid receptor 1 (TGR5) underscores bile acids’ dual roles as metabolic modulators and signaling molecules, impacting energy metabolism and immune functions (Chiang, 2017; Chiang et al., 2017).

### 2.2 Immunomodulation

#### 2.2.1 Effects on adaptive immunity

In adaptive immunity, bile acids regulate T and B cell responses by affecting antigen presentation and guiding lymphocyte differentiation (Fiorucci et al., 2025). For instance, 24-nor-UDCA (Ursodeoxycholic Acid, UDCA) modifies MHC-II expression patterns on macrophages and dendritic cells, consequently affecting T cell activation dynamics (Sombetzki et al., 2015), whereas chenodeoxycholic acid elevates MHC-I expression via PKC/PKA-dependent signaling. 3-oxolithocholic acid inhibits Th17 cell differentiation by directly binding to the nuclear receptor RORγt, effectively suppressing its transcriptional activity. Meanwhile, isoallolithocholic acid promotes the development of Foxp3^+^ regulatory T cells by increasing mitochondrial reactive oxygen species (ROS) production, which enhances epigenetic modifications supportive of Treg differentiation (He X. et al., 2025; Hang et al., 2019; Li et al., 2022). These findings highlight the roles of specific bile acid metabolites in fine-tuning immune cell differentiation through distinct molecular pathways.

Isodeoxycholic acid further amplifies peripheral Treg differentiation by diminishing DC immunostimulatory capacity, a mechanism potentially relevant to CMDs considering Tregs’ cardioprotective functions in myocardial infarction (Rizk et al., 2018). Beyond CD4^+^T cells, the regulation of CD8^+^T cells by bile acids is also closely related to CMDs. A recent study (Cong et al., 2024) identified via systematic screening that specific bile acid metabolites (e.g., deoxycholic acid, DCA) regulate CD8^+^ T cell function and identified related bile acid-producing microbiota (e.g., *Clostridium* spp., *Eubacterium* spp.).

#### 2.2.2 Effects on innate immunity

In innate immunity, BAs regulate macrophage polarization states and cytokine secretion profiles. Deoxycholic acidpotentiates pro-inflammatory M1 polarization through M2-muscarinic acetylcholine receptor (MAChR)/Src–TLR2–NF-κB/ERK/JNK pathway activation, whereas ursodeoxycholic acid (UDCA) favors anti-inflammatory M2 polarization (Zhou et al., 2021). TCDCA (Taurochenodeoxycholic acid, TCDCA) drives monocyte differentiation toward DCs with attenuated IL-12 production via TGR5-dependent signaling (Ichikawa et al., 2012). Significantly, BA-mediated NLRP3 (NOD-, LRR- and Pyrin Domain-Containing Protein 3, NLRP3) inflammasome activation constitutes a pivotal nexus between metabolic stress and chronic inflammation in CMDs. Elevated DCA (Deoxycholic Acid, DCA) and CDCA (Chenodeoxycholic Acid, CDCA) concentrations induce NF-κB priming and mitochondrial damage in macrophages, leading to NLRP3 inflammasome activation via the S1PR2–cathepsin B axis. Conversely, lithocholic acid (LCA) suppresses NLRP3 activation via protein kinase A (PKA)-mediated ubiquitination and proteasomal degradation. TGR5 signaling attenuates inflammasome-dependent inflammation, curtails pro-inflammatory cytokine release, and restricts macrophage uptake of oxidized LDL, thereby mitigating atherosclerotic plaque development (Moretti et al., 2018).

#### 2.2.3 The gut microbiota–bile acid–inflammation axis in CMDs

In cardiometabolic diseases (CMDs), bile acid (BA)-mediated immune regulation shows varied effects; while some bile acids like lithocholic acid (LCA) are anti-inflammatory, elevated levels of pro-inflammatory deoxycholic acid (DCA) and chenodeoxycholic acid (CDCA) activate the NLRP3 inflammasome, sustaining chronic inflammation (Chen et al., 2023; Hoang et al., 2024). Gut microbiota imbalance alters bile acid pools, increasing DCA and CDCA but decreasing LCA, which worsens inflammation through enhanced inflammasome activation and reduced NLRP3 degradation. This persistent inflammation disrupts metabolic balance and accelerates atherosclerosis, linking gut dysbiosis to CMD progression (Collins et al., 2023; Zhang et al., 2023).

## 3 Mechanistic roles of bile acids in cardiometabolic and cerebrovascular diseases

Bile acids are not merely digestive agents; they function as signaling molecules that regulate intricate metabolic and inflammatory pathways. Based on their core metabolic and immune roles, this section explores the gut microbiota–bile acid–immune axis, illustrating how microbiota-induced changes in bile acids contribute to the development of major cardiometabolic and cerebrovascular diseases.

### 3.1 Metabolic dysregulation in obesity and diabetes: the bile acid perspective

In obesity and diabetes, gut microbiota imbalance alters bile acid composition, reducing secondary bile acids and modifying FXR/TGR5 signaling. This disruption impacts glucose and lipid metabolism and initiates chronic low-grade inflammation. Bile acids play critical roles in regulating lipid metabolism and energy homeostasis. Their imbalance is closely linked to obesity and diabetes. A crucial mechanism is bile acid-induced activation of TGR5 receptors on intestinal L-cells, promoting GLP-1 secretion and improving insulin release and energy use. Disruption of the bile acid–TGR5–GLP-1 pathway contributes to the metabolic dysfunction and glucose intolerance typical of obesity and type 2 diabetes (Shapiro et al., 2018). Metabolomic analyses suggest that incipient BA metabolic alterations may heighten type 1 diabetes susceptibility, potentially through complex microbiota-BA-immune system interplay (Tyagi and Kumar, 2025). In type 2 diabetes, glycoursodeoxycholic acid (GUDCA) concentrations are significantly diminished. Experimental GUDCA supplementation ameliorates oxidative stress, enhances insulin sensitivity, and modulates glutathione and malondialdehyde levels. FXR activation lowers plasma glucose, improves insulin resistance, and reduces hepatic inflammation and fibrosis markers, whereas FXR deficiency impairs glucose tolerance (Mudaliar et al., 2013). High-fat diet models demonstrate that pathological BA accumulation accompanies cholesterol metabolic imbalance and systemic metabolic derangements. These findings establish the restoration of BA homeostasis as a promising therapeutic strategy for improving metabolic health and reducing cardiometabolic risks (Tyagi and Kumar, 2025). Collectively, these data highlight that restoration of bile acid homeostasis can re-balance the microbiota–BA–immune axis, improving insulin sensitivity and mitigating inflammatory signaling in metabolic disorders.

### 3.2 Bile acid control of cholesterol homeostasis and atherogenesis

In atherosclerosis, gut microbial imbalance promotes the generation of pro-inflammatory bile acids (e.g., DCA, CDCA), reinforcing vascular inflammation through NLRP3 activation and lipid accumulation in macrophages. FXR critically coordinates cholesterol and BA metabolism, partly through modulating SHP (Small heterodimer partner, SHP) and liver X receptor (LXR) pathways. BA-FXR binding upregulates SHP expression, suppresses cholesterologenic genes, and promotes hepatic cholesterol uptake (Mohammed and Zalzala, 2025). LXR activation enhances expression of cholesterol transporters including ATP-binding cassette transporter A1 (ABCA1) and scavenger receptor class B type I (SR-BI), which are indispensable for high-density lipoprotein (HDL) biogenesis and cholesterol efflux. BAs also modulate enzymes like cholesterol 7α-hydroxylase (CYP7A1), impacting cholesterol excretion and systemic homeostasis (Ge et al., 2019). Dysregulated BA metabolism accelerates macrophage foam cell formation, thereby potentiating atherogenesis. Consequently, targeting FXR/LXR signaling may benefit both cholesterol homeostasis and atherosclerosis management. Thus, FXR/TGR5 signaling represents a converging point where gut microbiota-derived bile acids modulate cholesterol metabolism, macrophage polarization, and vascular inflammation—core processes linking dysbiosis to atherogenesis.

### 3.3 Vascular inflammatory pathways modulated by bile acid

Gut microbiota-driven bile acid remodeling acts as an upstream modulator of vascular inflammation, where increased DCA/CDCA and decreased LCA amplify endothelial dysfunction via NLRP3 and NF-κB pathways. Vascular wall macrophages internalize modified low-density lipoprotein (LDL), initiating foam cell formation and plaque progression (Navratil et al., 2015). Macrophage TGR5 activation suppresses NF-κB-mediated inflammation, reduces foam cell generation, and limits oxidized LDL uptake (Wang et al., 2024). Diet-microbiota interactions significantly influence BA metabolism; for example, inulin elevates gut bacterial bile salt hydrolase activity, increasing circulating BA levels that activate FXR and upregulate interleukin-33, thereby triggering type 2 inflammation (Makki et al., 2023). Specific BAs including deoxycholic acid (DCA) and lithocholic acid (LCA) activate endothelial NF-κB and p38 MAPK signaling, inducing inflammatory responses and endothelial dysfunction. Among these, DCA enhances endothelial inflammatory responses by activating the NLRP3 inflammasome, while the weakened inhibitory effect of LCA on NLRP3 further exacerbates endothelial damage, collectively promoting early pathological changes in atherosclerosis. Intestinal FXR hyperactivation elevates circulating ceramides, exacerbating metabolic dysregulation, whereas GUDCA supplementation suppresses this axis, lowers serum ceramides, and reduces atherosclerotic burden in ApoE^−^/^−^ mice.

**Figure 1:**
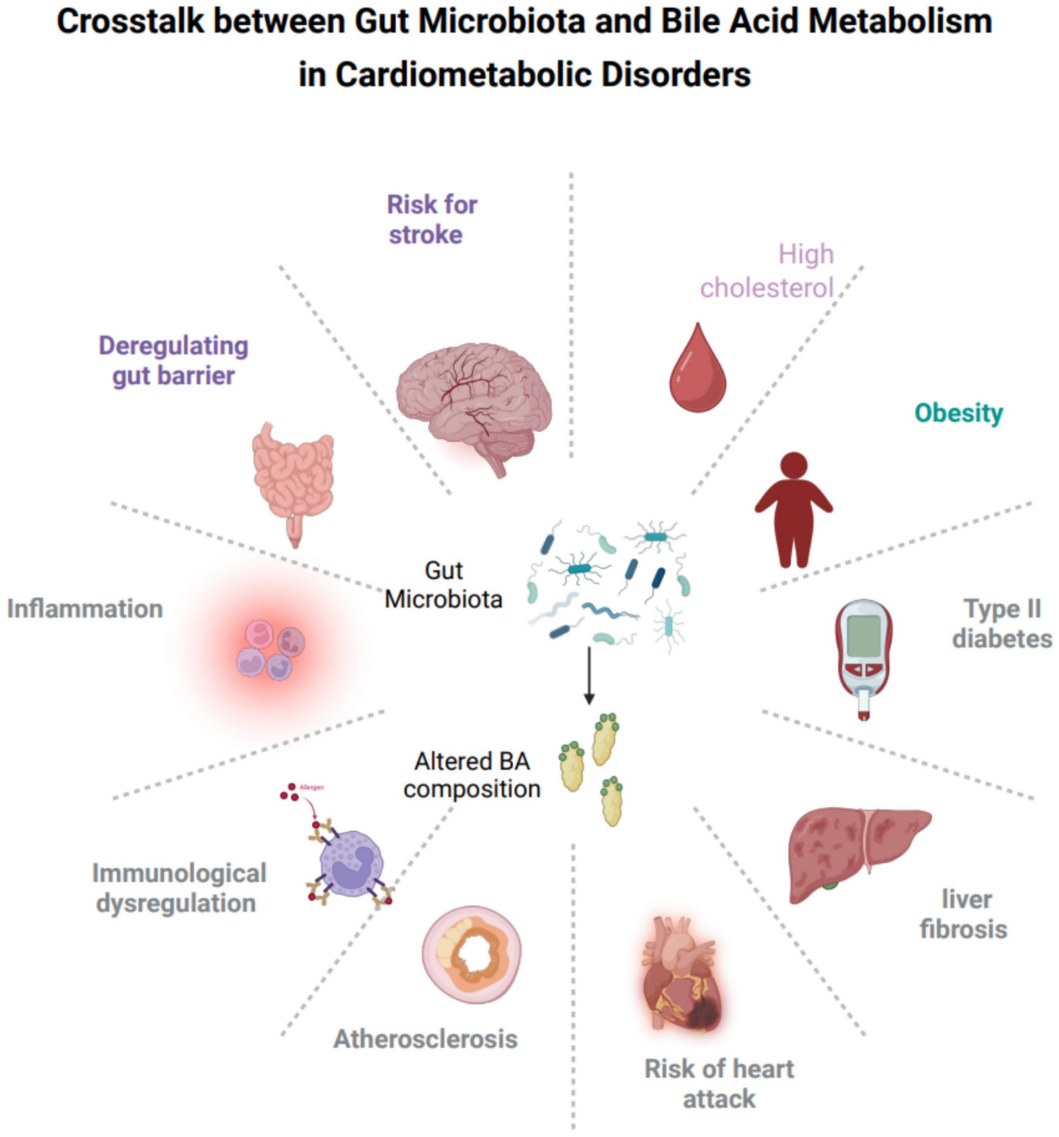
Crosstalk between gut microbiota and bile acid metabolism in cardimetabolic disorders. This figure summarizes how gut microbiota and bile acid composition interact to influence various cardiometabolic disorders. Alterations in these pathways are linked to obesity, type 2 diabetes, fatty liver, liver fibrosis, high cholesterol, atherosclerosis, heart attack risk, inflammation, immune dysregulation, and gut barrier changes. The diagram visually highlights the central regulatory role of gut microbiota–bile acid crosstalk in these disease processes. BAs, bile acids.

**Figure 2:**
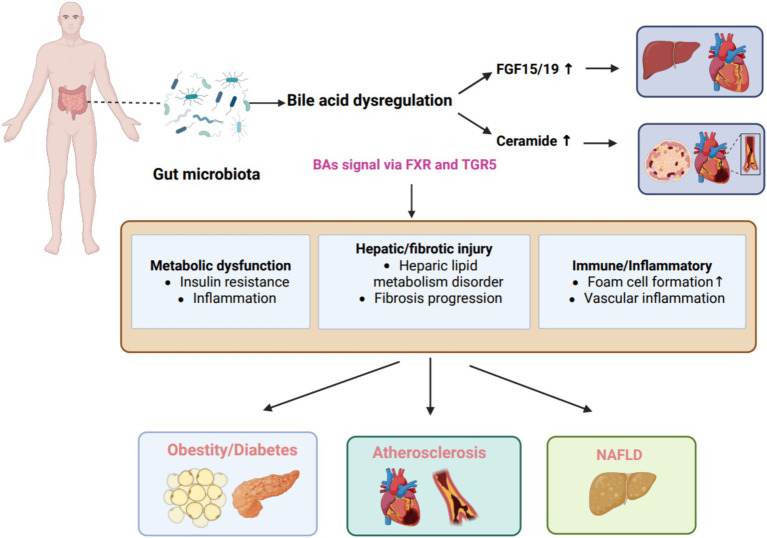
Bile acid dysregulation caused by gut microbiota imbalance. Dysregulated bile acids signal via FXR and TGR5 receptors, altering metabolic regulation, immune response, and inflammation. This activates the NLRP3 inflammasome, modifies cholesterol transport and ceramide metabolism, leading to insulin resistance, heightened inflammation, impaired GLP-1 (Glucagon-Like Peptide-1, GLP-1) pathway, increased foam cell formation and vascular inflammation, disordered hepatic lipid metabolism, and fibrosis progression, ultimately contributing to pathologies like obesity and diabetes. BAs, bile acids; FXR, farnesoid X receptor; TGR5, Takeda G protein-coupled receptor 5; NAFLD, Nonalcoholic fatty liver disease; FGF15\19, Fibroblast Growth Factor 19.

Cellular-level analyses reveal that BAs facilitate cholesterol transport from endocytic compartments to the endoplasmic reticulum for esterification, chylomicron packaging, and lymphatic secretion. FXR activation enhances reverse cholesterol transport, restricts intestinal cholesterol absorption, and attenuates NF-κB-dependent inflammatory signaling, conferring dual benefits for lipid regulation and vascular inflammation (Xu et al., 2016). Cumulatively, FXR and TGR5 signaling pathways represent compelling therapeutic targets for atherosclerosis prevention and management (Pathak et al., 2018). Recent studies increasingly highlight the complex, context-dependent duality of bile acid signaling. Systemic activation of FXR improves insulin sensitivity, regulates lipid metabolism, and reduces hepatic inflammation and fibrosis, thus exerting clear metabolic protective effects (Shapiro et al., 2018). Conversely, excessive intestinal FXR activation elevates circulating ceramide levels, fostering cholesterol metabolism imbalance and accelerating atherosclerosis development (Pathak et al., 2018). This paradox presents a critical challenge for developing bile acid receptor-targeted therapies, as tissue-specific and temporally controlled modulation may be required to maximize benefits while minimizing adverse cardiovascular outcomes. Together, these findings position the microbiota–BA–immune axis as a central inflammatory circuit bridging metabolic stress with endothelial injury and early atherogenesis (Figures 1, 2).

### 3.4 Biphasic roles of bile acids in heart failure: cardioprotection vs. myocardial injury

#### 3.4.1 Regulation of cardiac energy metabolism

In heart failure, altered gut microbiota composition and disrupted enterohepatic bile acid circulation reshape the cardiac immune–metabolic environment, influencing myocardial energy metabolism and remodeling. Bile acids play an essential role in maintaining myocardial energy supply, primarily through enhancing fatty acid β-oxidation and improving glucose utilization. FXR activation mediates these effects by increasing fatty acid oxidation efficiency in cardiomyocytes and promoting glucose uptake and utilization. This process supports cardiac energy homeostasis and contractile function (Hatamnejad et al., 2025). Additionally, bile acids modulate mitochondrial biogenesis and functional integrity, bolstering cardiac resilience to hypoxia and other stressors (Kapur et al., 2024). Consequently, maintaining an optimal bile acid profile appears essential for preserving cardiac metabolic health.

#### 3.4.2 Cardioprotective and toxic effects

Ursodeoxycholic acid (UDCA) exhibits anti-apoptotic and antioxidant effects, reducing oxidative stress and protecting cardiomyocytes from damage (Razavi et al., 2025). UDCA derivatives reduce pro-inflammatory cytokines and enhance peripheral perfusion in chronic heart failure patients. Moreover, the FGF15/19 axis, an entero-hepatic-cardiac signaling pathway that transmits bile acid–mediated metabolic cues from the intestine to the liver and the heart, serves as a critical mediator of systemic energy and lipid homeostasis. Dysregulation of this axis is directly associated with cardiac hypertrophy and metabolic remodeling. In contrast, hydrophobic bile acids such as LCA cause myocardial injury through intracellular calcium overload and elevated oxidative stress. Excessive bile acid accumulation also activates cardiac fibroblasts, accelerating myocardial fibrotic progression (Fanczal et al., 2020). These data underscore that bile acid composition and metabolic context are critical determinants of cardioprotective versus deleterious outcomes in heart failure (Aragón-Herrera et al., 2019).

#### 3.4.3 Bile acids and cardiac remodeling

Bile acid profile alterations strongly associate with ventricular hypertrophy and structural remodeling (Vasavan et al., 2018). TGR5 receptor activation inhibits fibroblast activation and decelerates remodeling processes. In preclinical heart failure models, bile acid receptor agonists improve cardiac metabolism, suppress inflammation, and attenuate ventricular hypertrophy, highlighting their therapeutic potential (Liu et al., 2013).

Overall, the dual cardioprotective and cytotoxic effects of bile acids reflect the systemic impact of the gut microbiota–BA–immune axis on cardiac metabolism, inflammation, and fibrosis progression.

## 4 Therapeutic potential of targeting bile acid pathways

Recent advances in bile acid research have paved the way for intervention strategies targeting these pathways in CMDs. This section reviews two key approaches: FXR/TGR5 agonists and microbiota-directed therapies, and combination drug strategies.

### 4.1 FXR and TGR5 agonists

FXR agonists like obeticholic acid (OCA) reduce hepatic inflammation, enhance insulin sensitivity, and attenuate fibrosis in preclinical and early-phase clinical trials (Ali et al., 2015). TGR5 activation promotes energy expenditure, improves endothelial function, and suppresses vascular inflammation, suggesting therapeutic utility for atherosclerosis and metabolic syndrome (Van Nierop et al., 2017). Nevertheless, adverse effects such as pruritus, dyslipidemia, and potential hepatotoxicity limit clinical application, driving the need for more selective or tissue-specific agents. New dual agonists targeting both FXR and TGR5 are being explored, with hopes of achieving stronger metabolic and anti-inflammatory effects while minimizing undesirable reactions (Miyata et al., 2021).

### 4.2 Gut microbiota modulation

As the gut microbiota heavily shapes bile acid metabolism, targeted interventions offer additional options for correcting bile acid imbalances. Methods such as probiotic or prebiotic supplementation and fecal microbiota transplantation can adjust bile acid profiles and promote effective FXR/TGR5 signaling, benefitting lipid and glucose control. Supplementing the diet with fermentable fibers or polyphenols may further modulate bile salt hydrolase activity, increase secondary bile acid production, and reduce overall inflammation.

## 5 Conclusions and future perspectives

Bile acid metabolism occupies a critical junction intersecting lipid regulation, immune modulation, and cardiovascular homeostasis (Bao-Anh et al., 2025). Although therapeutic bile acid pathway modulation shows considerable promise, clinical translation faces limitations including inadequate receptor selectivity (e.g., systemic FXR activation causing pruritus and dyslipidemia), adverse effect profiles (e.g., TGR5 agonism potentially exacerbating diabetes in obesity models), and individual response variability (divergent outcomes from genetic/dietary influences on microbial bile salt hydrolase activity) (Zhao et al., 2025). These constraints reflect fundamental controversies in bile acid biology—notably its context-dependent signaling duality, wherein intestinal FXR-ceramide axis activation improves metabolic parameters yet accelerates atherosclerosis, while TGR5 exerts both anti-inflammatory and profibrotic effects.

Addressing these challenges requires prioritizing: (1) Development of tissue-targeted nanodelivery systems to minimize off-target toxicity; (2) Elucidation of organ-specific mechanisms (e.g., spatiotemporal TGR5 regulation in myocardial fibrosis) and causal BA pool-immune microenvironment interactions during disease progression (e.g., secondary BA accumulation in heart failure); (3) Precise modulation of key regulatory nodes (e.g., GUDCA supplementation or microbial 7α-dehydroxylase inhibition) to restore metabolic-immune homeostasis (Jin et al., 2019). Furthermore, it should be acknowledged that many human studies on bile acids remain predominantly correlative, underscoring the importance of mechanistic investigations to establish causal relationships and validate therapeutic targets, while the integration of multi-omics approaches capturing inter-individual variations in bile acid metabolism and drug response holds promise for advancing more precise and effective cardiometabolic therapies.
